# Lessons from mouse models in the impact of risk factors on the genesis of childhood B-cell leukemia

**DOI:** 10.3389/fimmu.2023.1285743

**Published:** 2023-10-12

**Authors:** Ana Casado-García, Marta Isidro-Hernández, Silvia Alemán-Arteaga, Belén Ruiz-Corzo, Susana Riesco, Pablo Prieto-Matos, Lucía Sánchez, Isidro Sánchez-García, Carolina Vicente-Dueñas

**Affiliations:** ^1^ Experimental Therapeutics and Translational Oncology Program, Instituto de Biología Molecular y Celular del Cáncer, Consejo Superior de Investigaciones Científicas (CSIC)/Universidad de Salamanca, Salamanca, Spain; ^2^ Institute for Biomedical Research of Salamanca (IBSAL), Salamanca, Spain; ^3^ Department of Pediatrics, Hospital Universitario de Salamanca, Institute for Biomedical Research of Salamanca (IBSAL), Salamanca, Spain; ^4^ School of Law, University of Salamanca, Salamanca, Spain

**Keywords:** childhood leukemia, B-ALL, mouse models, risk factors, genetic predisposition, environmental factors

## Abstract

B-cell acute lymphoblastic leukemia (B-ALL) stands as the primary contributor to childhood cancer-related mortality on a global scale. The development of the most conventional forms of this disease has been proposed to be conducted by two different steps influenced by different types of risk factors. The first step is led by a genetic insult that is presumably acquired before birth that transforms a healthy cell into a preleukemic one, which is maintained untransformed until the second step takes place. This necessary next step to leukemia development will be triggered by different risk factors to which children are exposed after birth. Murine models that recap the stepwise progression of B-ALL have been instrumental in identifying environmental and genetic factors that contribute to disease risk. Recent evidence from these models has demonstrated that specific environmental risk factors, such as common infections or gut microbiome dysbiosis, induce immune stress, driving the transformation of preleukemic cells, and harboring genetic alterations, into fully transformed leukemic cells. Such models serve as valuable tools for investigating the mechanisms underlying preleukemic events and can aid in the development of preventive approaches for leukemia in child. Here, we discuss the existing knowledge, learned from mouse models, of the impact of genetic and environmental risk factors on childhood B-ALL evolution and how B-ALL prevention could be reached by interfering with preleukemic cells.

## 1 Introduction

B-cell Acute Lymphoblastic Leukemia (B-ALL), also known as B-cell precursor ALL, is one of the principal types of human leukemias. It is a clonal malignant disease that primarily affects children (1), characterized by the accumulation of blast cells that resemble the initial steps of normal B cell development.

B-ALL originates from a single cell and involves the abnormal enlargement of precursor B-cells, which exhibit phenotypic similarities to healthy precursor B-cells. There has been debate regarding the specific cell-of-origin for B-ALL, with some suggesting that it arises from a committed B cell (2). However, the exact origin of B-ALL is still a topic of ongoing discussion. Recent studies conducted in mouse models have provided insights into the development of B-cell leukemias showing that the restricted expression of an oncogene (*ETV6-RUNX1* or *BCR-ABL*) to the hematopoietic stem/progenitor cell (HSPC) compartment of mice is capable of inducing the disease (3–5). These studies indicate that genetic modifications can act in a “hit-and-run” manner on the cell-of-origin, establishing a tumor cell character. This unique mechanism of cell transformation involves an “epigenetic priming” process initiated by the initial genetic lesion. Interestingly, this initial hit may not be necessary for the subsequent evolution and persistence of the tumor, as the epigenetic priming becomes the driving force behind tumor development (6, 7).

Despite the molecular heterogeneity of B-ALL, there are common biological characteristics shared by a significant percentage of childhood B-ALL cases. Firstly, B-ALL leukemia in child exhibits a distinct age scattering, with a highest incidence between 2 and 5 years of age, followed by a decline in rates (8, 9). Furthermore, B-ALLs generally respond well to chemotherapy, leading to significant improvements in survival rates (reaching nearly 90%) among affected children over the past five decades (1). However, it is important to note that existing treatments are often associated with significant toxicity and morbidity (10) and between 10% and 20% of patients, who have achieved complete remission after initial treatment for ALL, will relapse and will have worse outcomes (11). Prior to being able to present novel therapeutic strategies for children, or ideally, institute preventive measures to hinder the onset of leukemia, it is imperative to comprehend the biological processes underlying the disease, and to this ambition, modelling the disease in mice is crucial.

**Figure 1:**
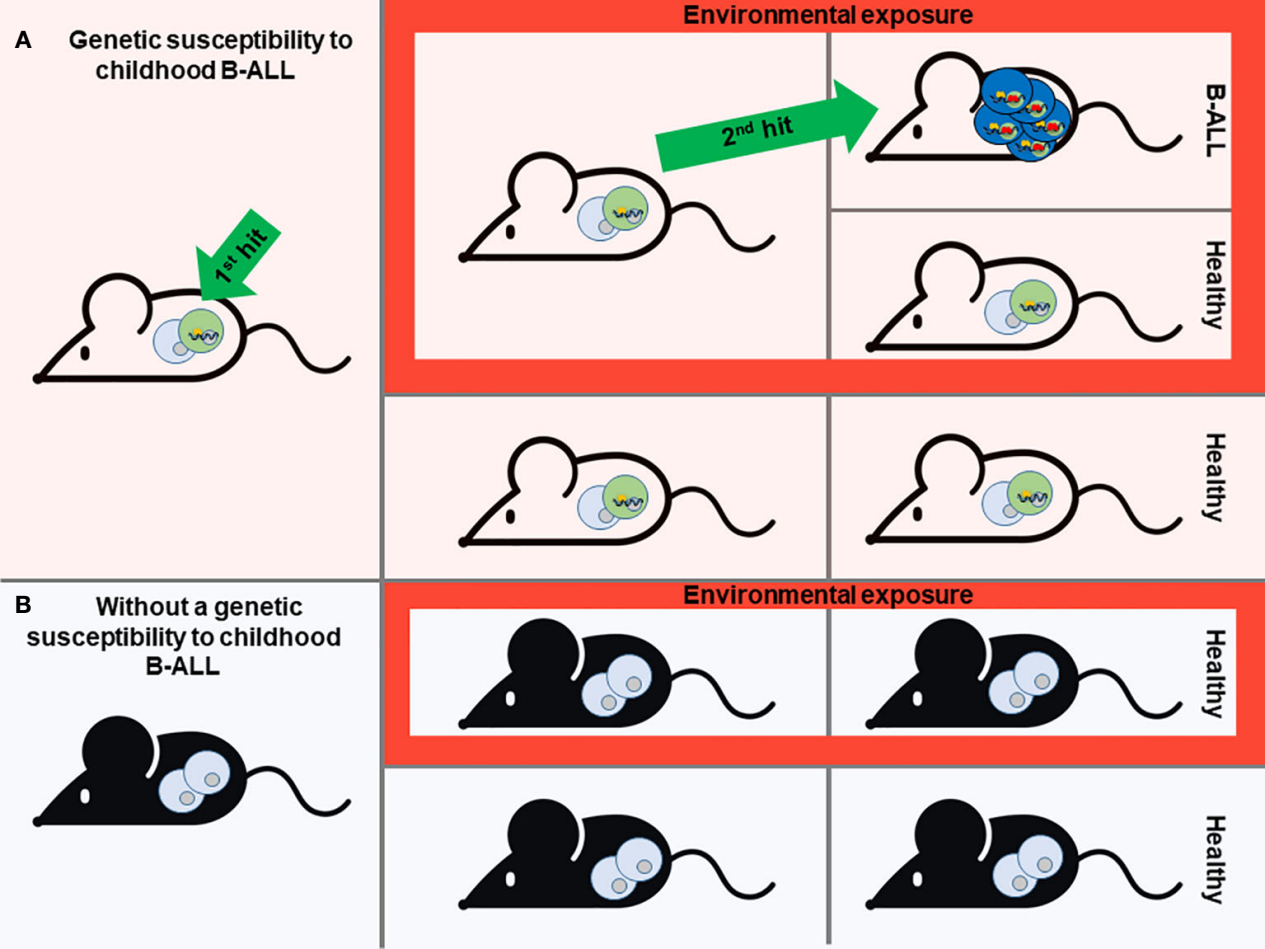
Modeling of Childhood B-ALL development. A prevailing theory for most B-ALL cases is the “Two-hit” model. This model suggests that the development of B-ALL involves two key genetic events. The first event, often acquired before birth (the first hit), initiates the formation of preleukemic cells. Subsequently, a second genetic mutation occurs after birth (the second hit), which drives the full development of B-ALL. Mouse models that mimic the stepwise progression of B-ALL are instrumental in identifying environmental and genetic factors that contribute to disease risk **(A)**. Based on this scenario, different genetic susceptibilities to childhood leukemia (first hit) have been replicated in mice, giving rise to preleukemic cells that are susceptible to transformation **(A)**. In the absence of this genetic susceptibility, mice will never develop B-ALL although they are exposed to environmental factors associated with this type of leukemia **(B)**. However, not all individuals who carry the genetic susceptibility to childhood B-ALL will ultimately develop the disease, despite exposure to environmental triggers. This phenomenon remains enigmatic **(A)**. Mouse modeling is essential to understand this issue and to identify environmental or other factors that have a direct association with the heightened susceptibility to this disease.

One of the most notable biological features of many childhoods B-ALL types is the presence of a latent silent preleukemic stage. During this phase, the initial event that triggers leukemia is present, yet the actual development of leukemia remains dormant and unapparent. This preleukemic situation can be found in up to 5% of healthy children, yet only a minority fraction (less than 1%) among the susceptible children will progress to develop B-ALL (12, 13). So, it is accepted that for the most common forms of B-ALL follow a “two-step” model of B-ALL development, from the preleukemic step to the leukemic one. It is likely that certain external or environmental factors play a role in triggering the progression from the preleukemic stage to full-blown disease (**Figure 1**). In recent decades, the incidence of B-ALL has shown an increase, which appears to be associated with modern lifestyle changes or exposures (14). Several epidemiological studies have shown that certain environmental factors, as is the case of exposure to infectious agents during infancy, are associated with childhood B-ALL (15–17). However, the practical prerequisites needed to obtain clear-cut solutions to the proposed epidemiological theories can only be met through experimentation in animal models. In this context, the creation of mouse models emulating the initiation of B-ALL has played a pivotal role in identifying the environmental and genetic elements that support disease risk.

*In vivo* research using mouse models incorporates all levels of organization relevant to the occurrence of the disease, ranging from the intricate structure of organs and their various cell types to the overall physiological condition of the organism. Furthermore, the genetic similarity among mice in these models provides scientists with precise control over genome alterations. These animal models have played a significant role in advancing our comprehension of the natural progression of B-ALL, spanning from the preleukemic phase to the totally transformed phase. In this context, some experimental models cited in this review have demonstrated both similarities in physical characteristics (phenotype) and genetic traits (genotype) with the human disease they are replicating. However, some of them are of limited value for environmental risk factors identification as the disease incidence cannot be consistently increased or decreased by exposure to those external agents that this disease is associated with.

## 2 Risk factors and B-ALL initiation

As we have already mentioned, leukemia stands as the primary contributor to childhood cancer-related deaths globally, and within this category, B-ALL ranks as the most prevalent subtype (11, 18). While the precise origins of acute leukemias remain unclear, it is widely acknowledged that several factors increase susceptibility to these blood disorders. These influential elements encompass both genetic and environmental components (19, 20).

The aforementioned “two-step” model of B-ALL development serves as the essential foundation for modelling the illness in mice. Consequently, the model should encompass the initial triggering event, which can either be acquired during prenatal development through somatic mutations or as a constitutional germline genetic variant. The prenatal origin of this disease is particularly true for germline mutations and inferred for somatic mutations based on studies from monozygotic twins carrying identical genetic alterations (21). While the second event can also be incorporated into mouse models, it holds greater significance to utilize mouse models that solely harbor the first oncogenic event. This approach enables the evaluation of potential risk factors that facilitate the natural occurrence of additional events. (**Figure 1**).

### 2.1 Genetic risk factors modelled in mice

Numerous pieces of evidence point to the involvement of genetic factors in the development of acute leukemias. Although the process of leukemogenesis remains partially incomplete, in recent years there have been substantial advancements in understanding the mechanisms responsible for the malignant conversion of hematopoietic precursor cells. However, despite these strides, much of this knowledge has yet to translate into direct benefits for patients. Utilizing mouse models that faithfully replicate the genetic predisposition to childhood leukemia has increased our knowledge of the malignant transformation mechanism behind this disease and can significantly contribute to unravelling the intricate connections between leukemia and environmental factors.

The genomic landscape of pediatric leukemia patients has undergone comprehensive characterization (22–25). In this context, in pursuit of comprehending the disease’s etiology, numerous mouse models have been created, each harboring distinct genetic alterations (germline or prenatally acquired somatic mutations) analogous to those found in humans (7).

An increasing array of genetic changes impacting B-cell transcription factors has been uncovered. These changes either directly trigger or make individuals more susceptible to the development of B-ALL. (22–24, 26–29). One illustrative instance is the *PAX5* gene, a pivotal transcription factor governing B-cell identity; approximately 30% of pediatric B-ALL cases exhibit somatic mutations or deletions impacting this gene (22, 24). Accordingly, several models have been developed altering the expression of *Pax5* in mice that subsequently develop B-ALL (30–36). Somatic alterations involving *Pax5*, emulated in mice (e.g. *PAX5-JAK2, PAX5-ELN* or *PAX5^ETV6/+^*), achieve almost complete penetrance of nearly 100% (33, 34, 36). Interestingly, germline *Pax5* mutations modeled in mice (e.g. Pax5^+/-^ mice) display an incomplete penetrance (32), mirroring the scenario observed in humans (26, 27, 29). Similarly, IL7R activating mutations (which is essential for lymphoid development) are also frequent in B-ALL. Mice harbouring these mutations have been made and acquire B-ALL with also a variable penetrance depending on the cell-of-origin in which the mutation is expressed (37–39). When the IL7R mutational activation occurred from the CLP stage and in homozygosis the incidence in nearly 100% (37).

Chromosomal translocation stands as one of the most prevalent genetic aberrations observed in B-ALL, with the *ETV6-RUNX1* fusion gene (t(12;21)(p13;q22)) being the most frequently encountered (40). While attempts to model the *ETV6-RUNX1* translocation in mice have been made, the majority of these models have failed to induce leukemia due to the expression of the fusion gene in committed B-cells (41–46). However, restricting the expression of *ETV6-RUNX1* to more immature, malleable hematopoietic cells, particularly murine stem cells, can lead to the development of childhood B-ALL under environmental pressures, such as common infection exposures (3). B-ALL instigated by either the *E2A-PBX1* (t(1;19)(q23;p13)) or the *BCR-ABL* (t(9;22)(q34;q11)) fusion genes has also been successfully modeled in mice (3, 47–51), albeit with varying penetrance. The discrepancy in penetrance observed among studies, despite expressing the same genetic alteration, relies on the fact that each study used different promoters and the cell of origin in which this promoter is active can differ a lot. The best mouse model will be the one that expresses the mutation in the right cell of origin and mimics the human pathology despite not having a complete penetrance.

The creation of these mouse models underscores the paramount importance of not only precisely identifying the genetic mutations existing in humans but also determining the specific cell-of-origin responsible for the emergence of B-ALL. This process aids in tailoring targeted interventions, ultimately culminating in the generation of an authentic and valuable mouse model for childhood B-ALL.

### 2.2 Environmental risk factors modelled in mice

In the most common forms of B-ALL, a genetic alteration, either acquired *in utero* or as a constitutional germline genetic variant, will give rise to the creation of preleukemic clones. These preleukemic cells could be present in healthy carriers for their whole life, but in fewer cases, further postnatal alterations will trigger the second hit and transform the preleukemic cells into leukemic ones (19, 52). There is a huge number of epidemiological studies on environmental/perinatal risk factors (e.g. exposure to common infections, low doses of ionizing radiation, pesticide exposure, living in proximity to nuclear facilities, maternal intake of fertility treatment, high birth weight (≥4000 g), caesarean delivery etc.) that are linked to ALL (53–58), but few of them are established as risk factors due to the lack of biological pieces of evidence.

There are only a few instances in which solid biological or mechanistic evidence supports the epidemiological connection between a risk factor and paediatric B-ALL. The first recognized environmental risk factor for childhood B-ALL is the exposure to higher levels of ionizing radiation (IR). The primary support for this correlation originates from studies involving atomic bomb survivors in Hiroshima and Nagasaki (59, 60), as well as research into the consequences of IR utilization for both therapy and diagnostics (61).

The second, and so far, the last environmental risk factor case with robust biological or mechanistic evidence, is the exposure to infectious agents and the role of immune function as a risk factor for B-ALL development. In the context of this multi-step disease, the second step occur perinatally or during infancy and can be prompted by infectious agents challenging the already dysregulated immune system due to the presence of a genetic predisposition, which constitutes the first step. In this scenario, the biological evidence that endorses the epidemiological association came from the use of mouse models that faithfully mimic B-ALL (3, 32). Two mouse models has been used to prove the interaction between infection and B-ALL development, the *Pax5^+/-^* and the *Sca1-ETV6-RUNX1* mice (3, 32) that replicate the initial phase responsible for the predisposition to B-ALL observed in certain human patients. Consistent with the "two-step" model, *Pax5^+/-^* and the *Sca1-ETV6-RUNX1* mice housed exclusively in a specific pathogen-free (SPF) environment never developed B-ALL despite harbouring the genetic predisposition; however, when these predisposed mice were moved to a conventional facility where they are exposure to infectious agents, 22% of *Pax5^+/-^* and 10% of *Sca1-ETV6-RUNX1* mice developed B-ALL (3, 32). These discoveries offer the initial scientific evidence that exposure to infections could serve as a catalyst for the development of B-ALL in individuals with genetic predispositions. Moreover, the specific genetic changes linked to the progression to leukemia in these mouse models closely resemble those observed in human patients (3, 26, 27, 29, 34), further supporting the idea that these models faithfully recapitulate the human pathology and how valuable they are to test environmental risk factors. In relation to this environmental role in leukemia development, mouse model studies must be very exhaustive when detailing the housing conditions to which the animals are exposed since this can greatly affect the final result and reproducibility.

However, not all B-ALL genetic predisposition situations adhere to this mechanism, as demonstrated by the situation of B-ALLs containing the *BCR-ABLp190* oncogene. In mouse models, the development of *BCR-ABLp190+* B-ALL occurs independently of infection (4), this aligns with the observation that in humans, this particular subtype of B-ALL is rarely seen in children (62, 63). Moreover, chromosomal translocations linked to distinct infant leukemias, such as *MLL*, appear capable of inducing full-fledged leukemic progression without the requirement of subsequent events. Instead, alternative molecular mechanisms seem to be employed to achieve these outcomes and seems to be independent of environmental factors (64–66).

## 3 Mechanisms of action of B-ALL risk factors

### 3.1 Epigenetic priming as cell transformation process

A recently accepted justification has shed light on the relationship between the distinct immunophenotype observed in childhood ALL (B- or T-ALL) and the precise underlying genetic disorder responsible for its development. B-ALL and T-ALL exhibit unique genetic alterations that are exclusive to each of these biological entities, resulting in a strong connection between genotype and phenotype. Traditionally, it was believed that the pivotal genetic event occurred in a committed B- or T-cell, explaining the correlation with the immunophenotype. However, current findings propose an alternative perspective, suggesting that the initial genetic disruption has the ability to induce genetic priming in the cell-of-origin, thereby dictating the definitive phenotype of the transformed cell. In this novel notion of the transformation mechanism, the first abnormal genetic alteration imposes a specific cell-differentiation program in the cancer cell-of-origin, determining whether the resulting tumor cells will exhibit a B-cell or T-cell phenotype. Importantly, the primary oncogenic modification, while essential for tumor beginning, becomes dispensable once the differentiation program is activated (3–5, 67). Consequently, in later stages of transformation, the oncogene loses its necessity and does not play a critical role in tumor progression. As a consequence, targeting this oncogene for therapies may not be effective since it lacks significance during the later phases of tumor development. This epigenetic reprogramming phenomenon has been demonstrated in various animal cancer models that closely resemble human disease quite well (6).

An open question is whether epigenetic priming may also respond to specific environmental factors in the context of particular genetic susceptibility. In this context, a “gene–environment interaction” will increase the sensitivity to a given environmental exposure as a result of which a second hit will appear in a preleukemic cell that will drive leukemia formation. Mouse models will be instrumental in deciphering this issue.

### 3.2 Immune stress

We have previously described that the presence of infectious stimuli promotes leukemia in genetically engineered murine models that faithfully recapitulate childhood B-ALL caused by specific genetic predisposition (*Pax5^+/-^* and *Sca1-ETV6-RUNX1* mice) (3, 32). The infection exposure promotes the immune stress capable of triggering preleukemia-to-leukemia conversion within an immune-dysregulated system. The immune stress is defined as the stress experienced during immune activation (e.g. as a result of exposure to infections) that boosts innate and adaptive immune responses. This includes changes in hematopoietic cell balance as well as local and systemic production of cytokines. Consequently, *Pax5^+/-^* mice exhibit a significant higher proportion of (pro and pre)-B and immature B cells in the bone marrow (BM), coupled with a reduced amount of total B cells in the peripheral blood (PB) (32). Interestingly, *Pax5^+/-^* BM precursors display a remarkable sensitivity to IL-7 cytokine withdrawal, underscoring a potential cell vulnerability. These findings imply that having only one functional copy of the *Pax5* gene (*Pax5* heterozygosity) promotes the emergence of an abnormal B-cell precursor compartment in the BM. This impairs their ability to develop into mature peripheral blood B cells. Moreover, the immune stress incited by infection stimuli further fosters the acquisition of secondary mutations within this vulnerable B cell population.

Similarly, in the *Sca1-ETV6-RUNX1* mouse model, exposure to common pathogens induces a temporal expansion of an aberrant B-cell precursor compartment within the BM (3). These amplified preleukemic cells display heightened expression of Rag1 and Rag2 (RAGs: recombination-activating genes, critical for generating mature B cells and T cells) consequently promoting the acquisition of secondary mutations (3).

However, this immune stress can be promoted not only by infectious stimuli but also by other mechanisms. An example of this is the gut microbiome dysbiosis. Recent data from both *Pax5^+/-^* and *ETV6-RUNX1+* mice show that the genetic predisposition is firmly associated with changes in the gut microbiome as the genetic predisposition in each case can be predicted just based on the murine gut microbiome composition, irrespective of whether the animals develop B-ALL or not (68). The gut microbiome might serve as an integration hub for environmental signals that interact with the immune system, consequently influencing the risk of developing B-ALL. Experiments conducted on predisposed mice have provided evidence that perturbation of the gut microbiome caused by antibiotic treatment promotes B-cell ALL development, even in the absence of infectious stimuli (68). This indicates a protective role for the gut microbiome and suggests that its dysbiosis could constitute another form of immune stress capable of triggering B-ALL within a genetically predisposed context.

As a result, we have learned from mouse models that different immunological stressors are linked to the conversion of preleukemic B cells to B-ALL. Recent experiments involving a murine model have dived deeper into this mechanism of immune stress, indicating that dysregulation of innate immune signaling within preleukemic precursor B cells plays a pivotal role in driving this immune stress, ultimately culminating in leukemia development (69). Specifically, *Myd88*, a central player in immune cell activation via Toll-like receptors (TLRs), undergoes downregulation in immune-stressed pre-malignant cells, and through an inflammation-dependent mechanism, contributes to the onset of leukemia (69).

To sum up, we have learned from murine models that some environmental factors stimulate immune stress that favors the full transformation of a preleukemic cell (a healthy cell with a genetic predisposition) by perturbing innate immune signaling regulation.

## 4 Targeting B-ALL promotion: toward B-ALL prevention

It is difficult to avoid exposure to common environmental factors such as exposure to infections, but eliminating the preleukemic cells susceptible to transformation becomes plausible to prevent the development of B-ALL.

**Figure 2:**
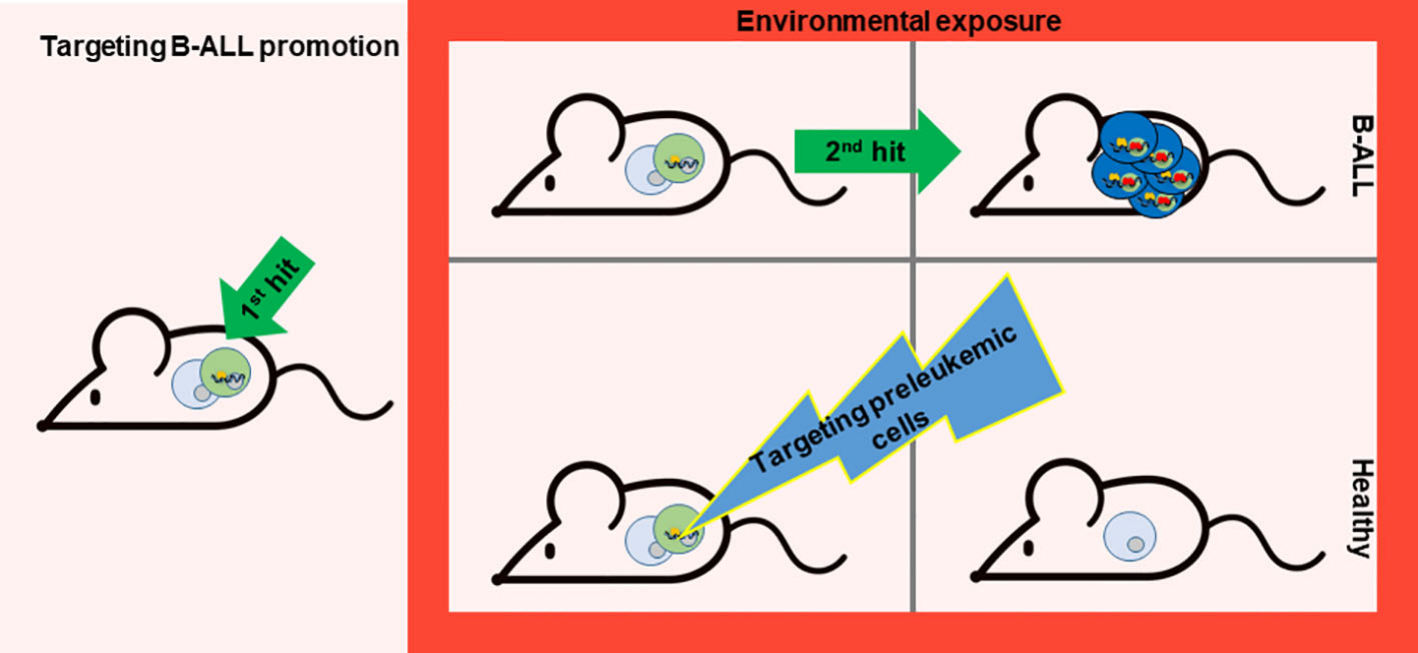
Targeting B-ALL progression. While evading exposure to environmental factors is challenging, the possibility of preventing B-ALL development lies in the potential elimination of preleukemic cells prone to transformation. Nonetheless, the key lies in discovering a method to specifically target these preleukemic cells. A recent study has paved the way for such a therapeutic approach (71). This proof-of-concept experiment was carried out in a mouse model that mirrors the characteristics of a leukemia-predisposition syndrome (Pax5^+/-^ mice), which develops B-ALL following exposure to common infections. Temporary administration of the Jak1/2 inhibitor ruxolitinib to Pax5+/− mice significantly diminishes the risk of leukemia occurrence, owing to the reduction of preleukemic cells.

A fundamental aspect to bear in mind toward B-ALL prevention is that the evolution from preleukemic cells to leukemic ones is led by the acquisition of secondary mutations as a result of environmental factors. The exposures do not facilitate the expansion of preleukemic clones already containing one or more second hits as has been demonstrated in previous mouse studies (32, 70). The second mutation appears just before B-ALL transformation, indeed if the first and the second hit are induced at the same time in the cell (e.g. *Pax5* and *Jak3* mutations), B-ALL development is immediate and as a consequence cannot be preventable (71). As the external factors initiate the emergence of these secondary mutations, this process could be prevented by eliminating the preleukemic cells before they fully transform (14, 72). In this regard, it is essential to explore methods for precisely targeting these preleukemic cells (**Figure 2**).

A recent study has opened the path for this kind of therapy. This proof-of-principle experiment has been conducted in a mouse model that recapitulates the phenotype of a leukemia-predisposition syndrome (*Pax5^+/-^* mice) that develop B-ALL upon exposure to common infections. Short-term administration of the Jak1/2 inhibitor ruxolitinib to *Pax5^+/−^* mice substantially decreases the likelihood of leukemia development by reducing the population of preleukemic cells (71). This approach is effective because *Pax5^+/−^* mice are especially dependent on the cytokine interleukin-7 (IL-7) for their survival, and impeding IL-7-induced signaling using the JAK1/2 inhibitor ruxolitinib led to augmented cell death *in vitro* (32). Based on these findings, *Pax5^+/−^* mice treated with ruxolitinib-containing chow for 28 days early in life eliminate preleukemic cells *in vivo* and thus avoid B-ALL development compared to mice fed with control chow (71) (**Figure 2**). All the mice in the experiment were exposed to common mouse pathogens thus the oncogenic environment persist, as this factor is difficult to avoid for prevention. Interestingly, *in vivo*, the administration of ruxolitinib exhibited a higher affinity for eliminating *Pax5^+/−^* B-cell progenitors compared to their wild-type (WT) counterparts (71). Thus, this is the first prevention approach for leukemia development as it is able to selectively eliminate preleukemic cells before transformation.

This strategy of eliminating preleukemic cells by the JAK-STAT inhibition using ruxolitinib indicates the potential utility of a similar strategy for children who are susceptible to developing B-ALL due to *PAX5* genetic susceptibility or other inherited mutations that render the preleukemic cells reliant on this signaling pathway. Given that some patients with germline mutations remain healthy into late age (26, 27, 29) the identification of biomarkers that can identify those healthy carriers that will develop leukemia will be essential to select those high-risk carriers who will benefit from interventional preventive therapies. In this regard, mouse models will be instrumental in identifying those biological markers that distinguish between those carriers that will develop the disease from the ones that will remain healthy despite carrying the genetic predisposition.

## 5 Future perspective

As mentioned earlier, there are various mouse models designed to replicate B-ALL through the manipulation of genetic mutations observed in humans. However, only a limited number of these models have proven effective in identifying environmental risk factors associated with the disease. This is primarily because most models do not inherently emulate the “multi-stage” progression characteristic of the majority of B-ALL subtypes, nor do they accurately represent the specific cellular origins where these genetic mutations occur.

Furthermore, B-ALL is marked by genetic diversity in the preleukemic cells’ predisposition, and the subsequent events that trigger disease progression also exhibit considerable variation. As a result, mouse models aiming to simulate this disease should take into account these crucial elements. B-ALL mouse models fulfilling these tasks will serve as valuable tools to assess preventive therapies to avoid leukemia development. Moreover, these models will offer valuable insights into the reasons behind the presence of healthy individuals who carry preleukemic cells but never progress to develop the disease. They will also aid in elucidating whether specific environmental factors, within the context of specific genetic susceptibilities, can initiate an epigenetic priming process that ultimately propels leukemia formation.

## Author contributions

AC-G: Writing – review & editing, Writing – original draft. MI-H: Writing – review & editing, Writing – original draft. SA-A: Writing – review & editing, Writing – original draft. BR-C: Writing – review & editing, Writing – original draft. SR: Writing – review & editing, Writing – original draft. PP-M: Writing – review & editing, Writing – original draft. LS: Writing – review & editing, Writing – original draft. IS-G: Writing – review & editing, Funding acquisition, Writing – original draft. CV-D: Writing – original draft, Writing – review & editing, Conceptualization, Funding acquisition.
